# External Therapy of Chinese Medicine for Herpes Zoster: A Systematic Review and Meta-Analysis

**DOI:** 10.1155/2022/3487579

**Published:** 2022-03-10

**Authors:** Liu Wu, Yong Chen, Tianming Man, Long Jin, Zhangmeng Xu, Lizeyu Lv, Jian Luo, Tianmin Zhu

**Affiliations:** ^1^School of Acupuncture and Tuina, Chengdu University of Traditional Chinese Medicine, Chengdu, China; ^2^School of Clinical Medicine, Chengdu University of Traditional Chinese Medicine, Chengdu, China; ^3^Department of Tuina, Hospital of Chengdu University of Traditional Chinese Medicine, Chengdu, China; ^4^School of Rehabilitation and Health Preservation, Chengdu University of Traditional Chinese Medicine, Chengdu, China

## Abstract

**Background:**

Herpes zoster (HZ) is a common skin disease that has a huge impact on the quality of life of sufferers. Antiviral therapy is a conventional treatment, but it still has limitations. This review evaluates the safety and efficacy of acupuncture in the treatment of HZ.

**Methods:**

We identified randomized controlled trials from multiple electronic sources (including Embase, PubMed, Cochrane, Web of Knowledge, China National Knowledge Infrastructure (CNKI), and China Biology Medicine Disc (CBM)) and reference lists of relevant articles and extracted data and assessed risk of bias (Cochrane's Risk of Bias tool). Pooled data are expressed as standardized mean differences (SMDs), with 95% confidence intervals (CI) (random-effects model).

**Results:**

We included 15 trials (1811 participants) comparing acupuncture to medicine. Ten studies involving 1424 patients provided these data for the meta-analysis. The results showed that acupuncture as a control group had a higher clinical cure rate than Western medicine therapy (*n* = 1424, 95% Cl 2.19–3.14, *I*^2^ = 0%). Eleven studies used the visual analog scale (VAS), but only nine provided specific data, which we used as a continuous variable for data extraction. The meta-analysis also showed an SMD of −2.64 (*n* = 646, 95% CI −3.79–1.48, *I*^2^ = 97%) which showed great heterogeneity. Meta-analysis showed a significant reduction in the incidence of PHN in those who received acupuncture compared to pharmacotherapy (OR = 0.35, 95% CI 0.04–2.86, *I*^2^ = 52%) which showed moderate heterogeneity. Economic indicators suggest that acupuncture costs less and has fewer adverse reactions.

**Conclusions:**

This review compares acupuncture therapy with conventional treatment and finds that the curative effects of acupuncture are exact, with fewer side effects. However, with the risk of bias and imprecision of the studies included, a concrete conclusion is difficult to draw. Thus, well-designed, rigorous studies are warranted in the future.

## 1. Introduction

Herpes zoster (HZ) (also known as shingles or zona) is a condition characterized by a painful rash and caused by the varicella-zoster virus (VZV) [1, 2], and the virus, also termed human herpes virus type 3 (HHV-3)], sometimes causes blistering rash over a limited part of the body [3, 4]. The typical case of chickenpox (varicella) is a child. On the contrary, the typical case of HZ is an elderly adult (the HZ incidence, which had increased from 2.5/1000 in 1993 to 7.2/1000 in 2016 among adults aged 35 years, the older the person, the higher the risk) [4, 5], who presents with a vesicular rash that is limited to a discrete area on one side of the body or face; the patient complains of pain, and recovery is sometimes associated with a chronic and intractable pain syndrome postherpetic neuralgia (PHN). Its after-effects are often expressed as PHN, which is the most distressing symptom [2, 5–9]. Approximately, 1 million people develop herpes zoster annually in America [10]. However, despite appropriate treatment with antiviral drugs, a substantial subset of HZ patients still develops complications, most notably postherpetic neuralgia (PHN) [11–13]. Pain is the major symptom that affects patients' quality of life (QoL) and is usually present across all phases of HZ disease [9, 14].

Shingles imposes a great financial burden on patients, especially those over 65 years of age [9]. For the moment, vaccines are the main way to prevent herpes [15, 16]. Nonetheless, specific vaccine recommendations should not divert attention from the urgent need to increase the currently inadequate uptake of HZ vaccine by adults. The debate about the age at which adults should get the HZ vaccine is still wide open [17, 18]. In addition, the effectiveness of HZ vaccine in preventing repeated episodes of HZ has not been demonstrated in clinical studies [19]. However, the patients at greatest risk are not currently eligible to receive the vaccine [17]. Patients with severe immunosuppression and individuals under the age of 40 who are at risk for vascular events have been shown to be the most at risk [20]. The main treatment for herpes zoster is antiviral therapy. About half of the patients were prescribed acyclovir, while the other half received valacyclovir, and a smaller number were prescribed famciclovir [21]. However, these antiviral drugs are excreted by the kidneys, and dosages must be adjusted to allow for renal insufficiency [22]. In addition, it includes only systemic corticosteroids and painkillers but with significant side effects [23, 24].

Acupuncture is an important therapy in traditional Chinese medicine (TCM). In clinical practice, acupuncture is used to treat various disorders, such as chronic pain syndrome, nausea, vomiting, and drug addiction [25]. In 2002, the World Health Organization (WHO) noted that acupuncture had an efficacy superior to that of control groups for up to 63 diseases, with significant efficacy for 28 of them [26]. Goldman et al. found that acupuncture could result in an abnormal increase of adenosine concentration in acupoint, which could activate local adenosine receptor A1 and produce a corresponding analgesic effect [27]. It is worth noting that this is just one of many studies on acupuncture analgesia [28]. Acupuncture therapy could accelerate healing and reduce pain of HZ [29]. Although previous reviews have described the safety and efficacy of acupuncture plus moxibustion for shingles, single studies have produced some positive results, and overall, there is insufficient evidence from high-quality studies reporting on objective outcomes [30], and the review did not follow the PRISMA principle.

The objective of the present systematic review was to identify and synthesize data from randomized controlled trials (RCTs) comparing acupuncture to usual care in adults diagnosed with HZ.

## 2. Method

The systematic review and meta-analysis are reported in accordance with the Prepared Items for Systematic Reviews and Meta-Analysis (PRISMA) guidelines [31]. The study has been registered on PROSPERO (CRD42020185674).

### 2.1. Search Strategy and Selection Criteria

We selected relevant studies published between Jan 1, 1950, and June 29, 2021, by searching Embase, PubMed, Cochrane, Web of Knowledge, China National Knowledge Infrastructure (CNKI), and China Biology Medicine Disc (CBM). The search was restricted to English- and Chinese-language studies. The search words were herpes zoster (e.g., herpes zoster, zoster, shingles, varicella-zoster virus, HZ, and VZV), acupuncture (e.g., needling, acupuncture, acupressure, electroacupuncture, cotton-moxibustion, tapping-capping, and fire needles). We considered all potentially eligible studies for review, irrespective of the primary outcome.

### 2.2. Inclusion and Exclusion Criteria

  Types of studies: all RCTs of acupuncture, including needling, acupuncture, acupressure, electroacupuncture, cotton-moxibustion, tapping-capping, and fire needles, for HZ were included, whether needling alone or not. Nonrandomized studies, quasitrials, and observational studies were excluded as well as animal studies, qualitative studies, letters, etc.
  Types of participants: clinical studies of adult participants with HZ were included. Studies were excluded if the condition being treated was PHN and complications of herpes zoster (zoster opthalmicus, zoster sine herpete, and visceral or disseminated zoster).
  Types of interventions: all types of acupuncture for HZ were included. Studies using interventions other than acupuncture that are applied with the same method in the acupuncture group and control group were included. However, dry needling not based on oriental medicine and meridian theory was excluded.
  Types of control groups: Western medicine for HZ was included. Sham acupuncture or traditional Chinese medicine alone, and not western medicine will be excluded in the control group.
  Types of outcome measures: in this study, we analyzed pain intensity, adverse reactions, recurrence rate of PHN, and QoL to evaluate the efficacy of the intervention group. The pain intensity was measured using the visual analog scale (VAS), McGill pain score, or other rating scales (e.g., verbal rating scale), and time to resolution of pain. We also analyzed economic indicators; Initial Zoster Impact Questionnaire, or other quality of life measures; therapeutic effective rate (TER), defined as a lesion improvement by 30% or more; and significant reduction in pain, according to a Chinese guideline [32].

### 2.3. Data Extraction

The retrieved literature was managed using EndNote X8 (Thompson Reuters). Two independent reviewers (LW and TMM) screened the title and abstract of the selected reference and included or excluded studies according to the eligibility criteria. Later, we subscribed the full text of the selected reference to determine the final selection. Disagreement was resolved by discussing with a third reviewer. Two reviewers independently read the full text of all of the studies and extracted data using a predefined form. Any disagreement was resolved by consultation between the two reviewers, and the final data were examined by another reviewer.

### 2.4. Assessment for Risk of Bias

Two reviewers (LJ and LW) independently evaluated the risk of bias of the final selected studies using the “risk of bias” tool of Cochrane Collaboration. This tool consists of seven domains, which are selection bias (random sequence generation and allocation concealment), performance bias (blinding of participants and personnel), detection bias (blinding of outcome assessment), attrition bias (incomplete outcome data), reporting bias (selective outcome reporting), and other sources of bias. Each study was evaluated as high, low, or unclear risk of bias on all of the domains, and the assessment criteria were based on the Cochrane handbook [33]. Disagreement between the two reviewers was settled by discussion.

### 2.5. Statistical Analysis

Statistics analysis was done using the Review Manager program (version 5.3 Copenhagen: The Nordic Cochrane Centre, The Cochrane Collaboration, 2014). All the studies were grouped and analyzed considering the outcome variable of each study and the characteristics of intervention. We analyzed pain intensity as continuous variables and were presented as standardized mean differences (SMDs) using inverse variance analysis. Other data are summarized using dichotomous variables. We used a random-effects model to combination treatment and also reduced the heterogeneity of the treatment-induced changes in outcomes in the comparator arm seen in the overall analysis. Heterogeneity between studies was evaluated by using 2 (chi-squared) test with a *p*value of *p* < 0 : 10 and I^2^ statistic. In case of substantial heterogeneity, the cause of heterogeneity was identified by analyzing subgroups. Publication bias analysis was not conducted in cases of less than 10 studies in a group.

## 3. Result

### 3.1. Description of Included Studies

**Figure 1:**
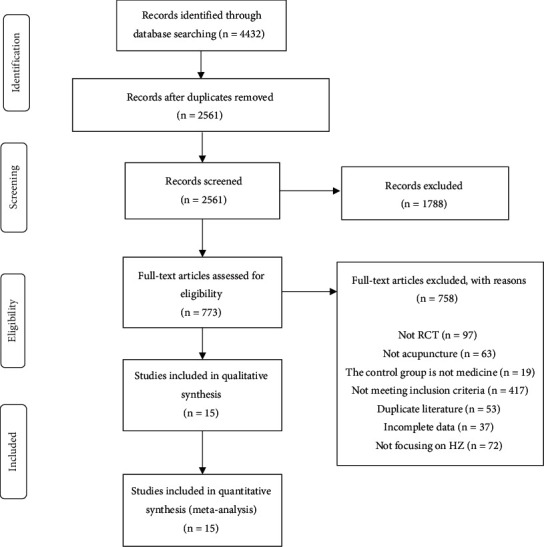
Flowchart of methods showing the detailed procedure for the inclusion or exclusion of studies.

We identified 4432 studies, of which 15 (with data for 1811 participants) were included in our analysis (Figure 1). Details of the 15 studies are summarized and shown in Table 1. The 15 trials were all published between 2011 and 2020 [34–48]. One RCT originates from Italy [43], while all remaining studies are from China. All RCTs adopted a parallel-group design. Ten involved two parallel-arm group designs [34–36, 38, 39, 41, 43–45, 47, 48], two involved five parallel-arm group designs [37, 40], one involved three parallel-arm group designs [42], and one involved four parallel-arm group designs [46]. The course of treatment was 10 days for nine articles [34, 35, 37–41, 44, 46], and two of the studies offered a course of 7 days [36, 47], one did not [45], and the other three offered a course of 14 days, 15 days, and 28 days. VAS was used as a pain rating scale in 11 studies [35, 36, 39, 41–44, 46–48], but one of the studies did not provide a specific mean or standard deviation [38], and one had different test scores on the VAS scale [38]. The intervention group in three studies was acupuncture combined with Western medicine [42, 46, 47]; the intervention group of 2 studies was acupuncture combined with traditional Chinese medicine [34, 45]; the intervention group of other studies was acupuncture (including electroacupuncture, cotton-moxibustion, tapping-capping, and fire needles).

### 3.2. Risk of Bias

**Figure 2:**
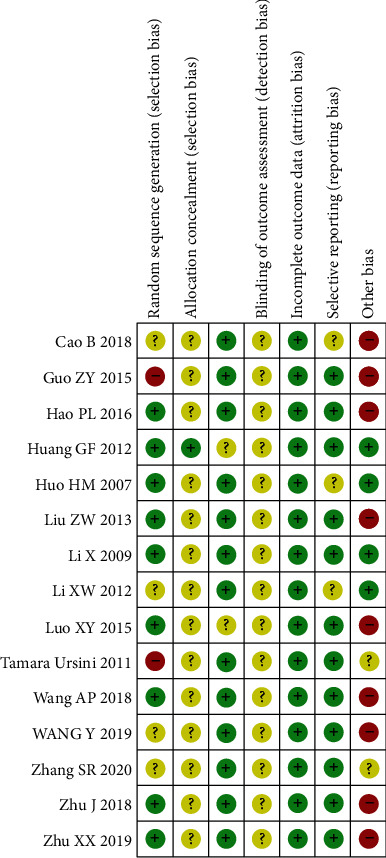
Risk of bias assessed using the Cochrane “risk of bias” tool.

The risk of bias was high in the included studies (Figure 2). All the studies used randomization, but only nine [36–40] of these studies reported using an appropriate method of random sequence generation, while two [35, 43] of these studies reported using inappropriate methods. Only one of the studies described the method for allocation concealment so that the risk of bias was evaluated as low [37]. The rest of the studies did not mention allocation concealment, and how the risk of bias was evaluated is unclear. Most of the studies did not perform blinding, but it does not affect the final outcome measurement resulting in a low risk of bias because both the physicians and the patients clearly knew which treatment was given. None of the studies described the method for blinding of the outcome assessment, resulting in a high risk of bias. All the studies did not mention detection bias and were classified as high risk. There was three studies that had dropouts [36, 37, 40], but it was still statistically significant, and the rest did not have incomplete data, so it was judged as low risk. Nine studies did not mention whether patients who had been previously treated with antiviral therapy had been excluded, which could cause bias and be judged as high risk [34–36, 41, 42, 44, 45, 47, 48].

### 3.3. Outcome

#### 3.3.1. Pain Intensity

**Figure 3:**
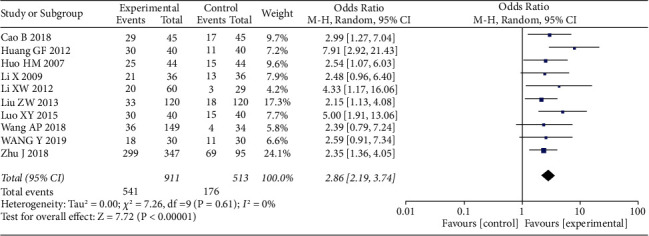
Forest plots with the random-effects model comparing acupuncture to medicine for HZ (dichotomous variable).

**Figure 4:**
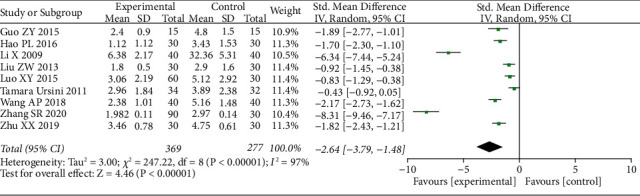
Forest plots with the random-effects model comparing acupuncture to medicine for HZ (continuous variable).

We extracted the pain data as dichotomous data and the complete disappearance of pain as the number of events. Ten studies [34, 37–42, 44, 45, 47] involving 1424 patients provided these data for the meta-analysis. The results showed that acupuncture as a control group had a higher clinical cure rate than Western medicine therapy (Figure 3) (*n* = 1424, 95% Cl 2.19–3.14, I^2^ = 0%). Eleven studies used VAS scales, but only nine [35, 36, 39, 41–44, 46, 48] provided specific data, which we used as a continuous variable for data extraction. The meta-analysis also showed a SMD of −2.64 (*n* = 646, 95% CI −3.79–1.48, *I*^2^ = 97%) which showed great heterogeneity (Figure 4).

#### 3.3.2. Incidence of PHN

Three studies noted the incidence of PHN [36, 41, 42]. Meta-analysis showed a significant reduction in the incidence of PHN in those who received acupuncture compared to pharmacotherapy (OR = 0.35, 95% CI 0.04–2.86, *I*^2^ = 52%) which showed moderate.

Heterogeneity: one of the studies was a three-arm experiment [42], which may have contributed to the heterogeneity: the Western medicine treatment group (group A), the Western medicine treatment plus mild reinforcing-reducing method group (group B), and the Western medicine plus cool-producing needling treatment group (group C). When subgroup analysis was performed when the study was removed, the heterogeneity results were significantly performed reduced (OR = 0.09, 95% CI 0.02–0.50, *I*^2^ = 0%).

#### 3.3.3. Adverse Reactions

Three studies reported on adverse events [38, 47]. One of them reported no adverse events occurred [47]. One case of upset stomach and one case of dizziness were reported in the control group of one study [38]. The adverse events were mild and the symptoms were relieved by expectant treatment. No adverse events were reported in any of the control groups. Other studies did not report any information on adverse events.

#### 3.3.4. Economic Indicators

One study reported on economic indicators [40]. The costs in descending order were as follows: Western medicine; cotton-sheet moxibustion; surrounding acupuncture plus electric acupuncture; tapping plus cupping; and puncturing with red-hot needles. Compared with Western medicine, puncturing with red-hot needles can save RMB 149.92 yuan, surrounding acupuncture plus electric acupuncture can save RMB 133 yuan, tapping plus cupping can save RMB 148.12 yuan, and cotton-sheet moxibustion can save RMB 117.16 yuan.

## 4. Discussion

The findings from this systematic review suggest potential benefits from acupuncture in reducing pain intensity, incidence of PHN, and costs. Our analyses showed high heterogeneity, in particular, VAS scores were analyzed as continuity variables. This was likely due to the poor methodological quality of some included studies, a relatively small sample size, and clinical heterogeneity. When we extracted the data using pain intensity (complete disappearance of pain as the number of events) as a dichotomous variable, no heterogeneity was shown, indicating that acupuncture has a significant advantage over drugs in pain relief. The therapeutic effective rate has not been validated and should be interpreted with caution. There is no consensus on the definition of PHN, but pain persisting three months after the resolution of the rash is generally accepted as the clinical definition and is used for research purposes [30]. Only three of the studies included in this review mentioned the incidence of PHN, due to the small number of included patients and moderate heterogeneity after analysis, the possibility of directly reducing the incidence of PHN needs to be carefully explained. It is worth mentioning that one study mentioned economic indicators that showed that acupuncture was cheaper than drug therapy.

HZ is a common, painful, and debilitating condition caused by a reactivation of VZV from a latent infection of sensory ganglia [14]. Approximately, one in three persons will develop HZ during their lifetime [49]. VZV infection is an absolute prerequisite for HZ, and the VZV-specific cell-mediated immune response decreases with advancing age, so older people are more likely to get HZ [50]. The virus can lie dormant for decades in the dorsal root ganglion until it is stimulated to reactivate and reproduce down the path of the nerve to the surface of the skin. The stimulation for viral reactivation is quite variable and includes stress, immunocompromised states, severe illness, and use of corticosteroids [51]. For the time being, the combination of antiviral therapy and analgesic drugs is the main intervention in the treatment of herpes zoster. When conventional treatment fails, other options are considered, for example, corticosteroids, anticonvulsants (e.g., gabapentin or pregabalin), tricyclic antidepressants (e.g., nortriptyline or desipramine), or neural blockade can be considered [22]. However, antivirals must be administered within 72 hours of symptom onset, and there is insufficient evidence to demonstrate a benefit in preventing PHN [52].

Acupuncture is one of the commonly used alternative therapies [53]. At present, the mechanism of action of acupuncture is still unclear, the commonly accepted consensus is that acupuncture triggers systemic responses, including responses in the nervous system, by physically stimulating specific acupoints on the surface of the human body, thereby regulating human body functions to eventually achieve a therapeutic effect [28]. The publication of various controlled trials has shown that acupuncture has a significant effect on pain syndrome such as acupuncture for acute and chronic low back pain, knee osteoarthritis, headache, myofascial pain, neck pain, and fibromyalgia [54]. Extensive research has shown that acupuncture analgesia may be initiated by stimulation, in the muscles, of high-threshold, small-diameter nerves [55]. These nerves are able to send messages to the spinal cord and then activate the spinal cord, brain stem (periaqueductal gray area), and hypothalamic (arcuate) neurons, which, in turn, trigger endogenous opioid mechanisms [56].

It is meaningful enough that acupuncture is as effective as, if not better than, the common treatment strategies for HZ such as medication and physical therapies. Since there were no serious adverse effects from acupuncture within the studies included in this analysis, acupuncture treatment can be considered for HZ. Moreover, HZ has a greater economic burden on patients, and the cost of acupuncture treatment is lower. Acupuncture may be expected to decrease pain of HZ even more. However, since the included studies were small and the risk of bias was high, further larger-scale studies of higher quality design are needed.

## 5. Conclusion

This review compares acupuncture therapy with conventional treatment and finds that the curative effects of acupuncture are four exact, with fewer side effects. However, with the risk of bias and imprecision of the studies included, a concrete conclusion is difficult to draw. Thus, well-designed, rigorous studies are warranted in the future.

## Figures and Tables

**Table 1:** Characteristics and intervention details of the included trials.

First author, publication year	Mean age (Mean ± SD)	Treatment duration/course of disease (Mean ± SD)	Design (no. of subjects)	Acupuncture points (intervention group)	Comparison (medicine)	Outcome reported
Cao B, 2018	IG: 43.6 ± 2.1 CG: 42.8 ± 2.3	10 days/(IG: 3.4 ± 0.5, CG: 3.6 ± 0.5)	2 parallel arms: TCM + AC + CM (44); WM (44)	Jiaji (EX-B2), Zhigou (SJ6), Yanglingquan (GB34), and Yinlingquan (SP9)	Acyclovir, vitamin B1, and ibuprofen	Comparison of therapeutic effect
Guo ZY, 2015	IG: 38.2 ± 5.6 CG: 37.3 ± 5.0	10 days/(not specified)	2 parallel arms: FN + AC (15); WM (15)	Baihui (DU20), Yintang (DU29), Shuaigu (GB8), Hegu (LI4), and Waiguan (SJ5)	Valaciclovir hydrochloride, mecobalamin, and vitamin B1	VAS, comparison of time of blister alleviation, and satisfaction survey
Hao PL, 2018	IG: 53 ± 15 CG: 53 ± 16	7 days/(IG: 2.8 ± 1.9, CG: 3.1 ± 1.8)	2 parallel arms: TC (30); WM (30)	Ashi point	Valaciclovir hydrochloride, vitamin B1, and vitamin B12	VAS, routine blood test, and follow-up
Huang GF, 2012	IG1: 44.1 ± 16.2 IG2: 46.7 ± 13.2 IG3: 44.9 ± 14.4 IG4: 47.3 ± 13.1 CG: 46.0 ± 14.7	10 days/(IG1: 3.6 ± 1.8 IG2: 3.1 ± 1.7 G3: 3.8 ± 1.8 IG4: 3.9 ± 2.0 CG: 3.3 ± 1.7)	5 parallel arms: AC + EC (35); CM (34); FN (42); TC (38); WM (34)	Ashi point, Jiaji (EX-B2), Zhigou (SJ6), and Houxi (SI3)	Valaciclovir hydrochloride and vitamin B1	Comparison of time of blister alleviation, VAS, and comparison of therapeutic effect
Huo HM, 2007	IG: 48.45 ± 13.12 CG: 47.32 ± 11.29	10 days/(IG: 3.05 ± 1.04, CG: 2.95 ± 1.74)	2 parallel arms: TC (120); WM (120)	Ashi point, Quchi (LI11), Hegu (LI4), Yinlingquan (SP9), and Taichong (LR3)	Acyclovir tablet and aciclovir cream	Comparison of therapeutic response time, comparison of the therapeutic effect, and adverse reaction
Li X, 2009	IG: 46.36 ± 10.21 CG: 47.79 ± 9.43	10 days/(not specified)	2 parallel arms AC + EC (40); WM (40)	Ashi point, Jiaji (EX-B2), Zhigou (SJ6), and Houxi (SI3)	Valaciclovir hydrochloride and vitamin B1	VAS, comparison of the therapeutic effect, and comparison of the crust formation
Li XW, 2012	IG1: 43.76 ± 15.43 IG2: 46.98 ± 13.61 IG3: 45.20 ± 15.06 IG4: 44.33 ± 15.07 CG: 46.51 ± 15.30	10 days/(IG1: 6.15 ± 4.11 IG2: 5.56 ± 3.14 IG3: 5.63 ± 2.70 IG4: 5.77 ± 3.05 CG: 5.24 ± 2.52)	5 parallel arms: AC + EC (98); CM (100); FN (97); TC (96); WM (98)	Jiaji (EX-B2), Zhigou (SJ6), Houxi (SI3)	Valaciclovir hydrochloride and vitamin B1	Comparison of the therapeutic effect, comparison of the cost
Liu ZW, 2013	IG: 44 ± 21 CG: 46 ± 18	10 days/(3.0 ± 1.5	2 parallel arms: AC + CM (30); WM (30)	Ashi point, Zusanli (ST36), and Guanyuan (RN4)	Famciclovir tablet, nimesulide dispersible tablets, and vitamin B1	VAS, QS, comparison of therapeutic response time, and recurrence PHN
Luo XY, 2015	IG1: 68.2 ± 3.8 IG2: 67.9 ± 4.1 CG: 66.7 ± 3.9	14 days/(IG1: 18.1 ± 8.0 IG2: 17.3 ± 8.4 CG: 17.6 ± 8.5)	3 parallel arms:AC + WM (30); AC + WM (30); WM (29)	Taichong (LR3), Zhigou (SJ6), Yanglingquan (GB34), Yinlingquan (SP9), Jiaji (EX-B2), and Xingjian (LR2)	Valaciclovir hydrochloride, Methycobal, pregabalin capsule, meloxicam tablets, and tramadol hydrochloride sustained release tablets	Comparison of the CD4+ level, VAS, recurrence PHN
Tamara ursini, 2011	IG: 65.5 ± 12.8 CG: 67.1 ± 12.8	28 days/(not specified)	2 parallel arms: AC (34); WM (32)	Zhongwan (CV12), Guanyuan (CV4), Quchi (LI11), Hegu (LI4), Neiting (ST44), Xuehai (SP10), Xingjian (LR2), and Neiguan (PC6)	Pregabalin, chirocaine, buprenorphine, oxycodone, and paracetamol	VAS, MPQ, recurrence rate of PHN, the total pain burden during the 12-month follow-up of the cohort study, and the incidence of treatment-related serious adverse events during treatment
Wang AP, 2018	Not specified	10 days/(not specified	2 parallel arms:AC + TC (40); WM (40)	Ashi point, Zhigou (SJ6), Dazhui (DU14), Hegu (LI4), and Fengchi (GB20),	Valaciclovir hydrochloride, cobamamide, and buprenorphine	VAS and comparison of clinical efficacy
Wang Y, 2019	IG: 72.2 ± 4.1 CG: 71.8 ± 4.3	Not specified	2 parallel arms:AC + TCM (36); WM (36)	Jiaji (EX-B2), Neiting (ST44), Yanglingquan (GB34), Yinlingquan (SP9), Zusanli (ST36), and Xingjian (LR2)	TDP irradiation, aspirin, and valaciclovir hydrochloride	Comparison of pain relief and comparison of relief of herpes
Zhang SR, 2020	IG1: 48.27 ± 2.16 IG2: 48.29 ± 2.14 IG3: 48.30 ± 2.13 CG: 48.31 ± 2.51	10 days/(not specified)	4 parallel arms: AC + WM + EA (30); AC + WM + EA + TC (30); AC + WM + EA + CM (30); WM (30)	Ashi point, Jiaji (EX-B2), Zhigou (SJ6), and Houxi (SI3)	Valaciclovir hydrochloride and vitamin B1	Comparison of relief of herpes, VAS, WHOQOL-100, and adverse reaction
Zhu J, 2018	IG: 46.34.2 ± 12.54 CG: 45.92 ± 13.1	7 days/(IG: 3.65 ± 1.658, CG: 3.64 ± 1.538)	2 parallel arms: AC + TC + WM (45); WM (45)	Ashi point, Zhigou (SJ6), Jiaji (EX-B2), and Houxi (SI3)	Valaciclovir hydrochloride and vitamin C	VAS and comparison of clinical efficacy
Zhu XX, 2019	IG: 47 ± 13 CG: 44 ± 15	15 days/(IG: 3.9 ± 1.6, CG: 3.7 ± 1.7)	2 parallel arms: AC (30); WM (30)	Ashi point	Valaciclovir hydrochloride dispersible tablets, mecobalamin tablets, and vitamin B1	Comparison of relief of herpes, VAS, serum immune-related factors (IgG, IgM, IgA), and serum inflammatory factors (IL-4, IL-17, TNF-*α*, TGF-*β*1) were observed before and after treatment in the two groups

IG: intervention group; CG: control group; AC: acupuncture; EA: electroacupuncture; CM: cotton-moxibustion; TC: tapping-capping; FN: fire needle; WM: Western medicine; VAS: visual analog scale; QS: sleep quality score; PHN: postherpetic neuralgia; MPQ: McGill Pain Questionnaire.

## Data Availability

All data generated or analyzed during this study are included in this published article. The datasets used or analyzed during the current study are available from the corresponding author on reasonable request.
